# The Sealing Zone in Osteoclasts: A Self-Organized Structure on the Bone

**DOI:** 10.3390/ijms19040984

**Published:** 2018-03-26

**Authors:** Jiro Takito, Satoshi Inoue, Masanori Nakamura

**Affiliations:** Department of Oral Anatomy and Developmental Biology, School of Dentistry, Showa University, 1-5-8 Hatanodai, Shinagawa, Tokyo 142-8555, Japan; s-inoue@dent.showa-u.ac.jp (S.I.); masanaka@dent.showa-u.ac.jp (M.N.)

**Keywords:** actin polymerization, actin wave, Arp2/3, integrin, invadopodia, osteoclasts, plasma membrane, podosome, sealing zone

## Abstract

Osteoclasts form a specialized cell–matrix adhesion structure, known as the “sealing zone”, during bone resorption. The sealing zone is a dynamic actin-rich structure that defines the resorption area of the bone. The detailed dynamics and fine structure of the sealing zone have been elusive. Osteoclasts plated on glass do not form a sealing zone, but generate a separate supra-molecular structure called the “podosome belt”. Podosomes are integrin-based adhesion complexes involved in matrix adhesion, cell migration, matrix degradation, and mechanosensing. Invadopodia, podosome-like protrusions in cancer cells, are involved in cell invasion into other tissues by promoting matrix degradation. Both podosomes and invadopodia exhibit actin pattern transitions during maturation. We previously found that Arp2/3-dependent actin flow occurs in all observed assembly patterns of podosomes in osteoclasts on glass. It is known that the actin wave in *Dictyostelium* cells exhibits a similar pattern transition in its evolution. Because of significant advances in our understanding regarding the mechanism of podosomes/invadopodia formation over the last decade, we revisited the structure and function of the sealing zone in this review, highlighting the possible involvement of self-organized actin waves in the organogenesis of the sealing zone.

## 1. Introduction

Osteoclasts are giant multinucleated cells responsible for bone resorption. When osteoclasts resorb bone, they form the sealing zone, a bone-anchored adhesion structure between the bone and the osteoclasts. The sealing zone demarcates the area of bone resorption from the rest of the environment. The exact structure and dynamics of the sealing zone have been elusive, owing to a lack of techniques that enable high-resolution in vivo imaging. Osteoclasts plated on glass form a podosome belt (also called the actin ring), one of the patterns of the podosomes. The podosome is an actin-rich matrix-anchored complex found in various cells, including macrophages, dendritic cells, and osteoclasts [1,2]. In the last decade, intensive in vitro studies have revealed the unique characteristics of podosomes: their detailed structure in 2-D, their dynamics, matrix degradation activities, and mechanosensitive behaviors [3,4,5]. We recently found that retrograde actin flow occurs in a podosome-derived structure within osteoclasts [6]. This information has shed light on the nature of the sealing zone of osteoclasts. Because we have already reviewed the role of actin flow in the podosome field elsewhere [7], the present study concentrates on the role of actin waves in the organogenesis of the sealing zone. Traditionally, the unique actin-rich adhesive structures in normal cells have been called podosomes, whereas in cancer cells, they are known as invadopodia. Because many podosome-like or invadopodium-like structures are present in various types of cells, the term “invadosomes” has been proposed as generic nomenclature [2]. However, in this review, we follow the traditional naming to clearly distinguish between the specific structure and function within a given cell.

## 2. Bone Resorption by Osteoclasts

**Figure 1:**
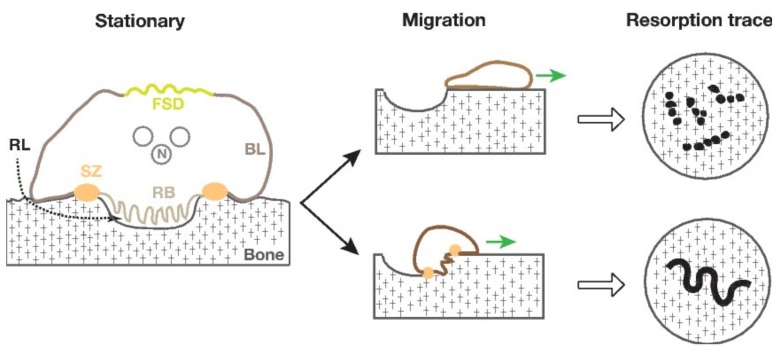
Polarization of osteoclast during bone resorption. The scheme on the left shows a cross section of the resorbing osteoclast in the stationary mode. The polarization of an osteoclast compartmentalizes its plasma membrane on the bone [9]. The resorption lacune is the space enclosed within the sealing zone, the ruffled border membrane, and the bone. The stationary resorbing osteoclast transits into one of the two migratory modes. The osteoclast stops bone resorption and migrates on bone (upper panel in the middle), or continues bone resorption in the migratory mode (lower panel in the middle). Green arrows indicate the direction of movement. The former mode of osteoclasts forms pit-type resorption traces (upper panel in the right), while the latter produces trail- or trench-type resorption traces (lower panel in the right) [14,15]. The right panels show the resorption traces in a bird’s eye view. BL, basolateral membrane; FSD, functional secretory domain; N, nucleus; RB, ruffled border membrane; RL, resorption lacune; SZ, sealing zone.

Bone resorption by an osteoclast accompanies the sequential reorganization of its cellular components, leading to the polarization of membrane domains (Figure 1) [8,9]. Firstly, an osteoclast forms the sealing zone through which the cell adheres to the bone surface and defines the area for bone resorption. The establishment of the sealing zone is followed by the formation of two new membrane domains: the ruffled border membrane inside the sealing zone and the functional secretory domain (FSD) at the opposite side of the bone. For the accomplishment of bone degradation, an osteoclast secretes protons, to dissolve hydroxyapatite, and proteases, to digest the organic materials of bone through the ruffled border membrane [10,11]. The digested materials of bone are discharged from the FSD via the transcytotic route [12,13]. Thus, an osteoclast temporarily creates three new membrane domains for bone resorption. In the classical model, an osteoclast enters into the migration mode after bone resorption, during which the cell loses its sealing zone. However, resorption traces made by osteoclasts suggest that some osteoclasts can resorb bone during migration [8]. Recent studies reveal the characteristics of these osteoclasts set in the migration mode with the sealing zone [14,15]. It is therefore apparent that osteoclasts exhibit remarkable plasticity, allowing them to reconstruct their cellular substructure during bone resorption. The detailed properties of the sealing zone and the compartmentalization of the ventral membrane of osteoclasts on bone will be discussed in Section 4.3 and Section 6.2, respectively. The sealing zone is essential for osteoclastic bone resorption. Src-deficient mice exhibit osteopetrosis in spite of abundant osteoclasts in the bone. These osteoclasts are dysfunctional because of a defect of the ruffled border membrane [16,17]. So, we can separate the bone-resorbing activity from the differentiation of osteoclasts.

## 3. Structure of the Sealing Zone

### 3.1. Podosomes and the Sealing Zone

The sealing zone has originally been identified as the adhesion structure of chondroclasts in mineralized cartilage [18]. Because the sealing zone has no cytoplasmic organelles and has a uniform appearance when observed using electronmicroscopy, it is also called the “clear zone”. In culture, isolated chicken osteoclasts adhere to glass via a continuous paramarginal area containing punctate F-actin rich structures called podosomes [19]. Since podosomes’ identification, the paramarginal area, also called “podosome belt” or “actin ring”, has been a subject of intense research. Live-cell imaging of an in vitro osteoclast model, namely, RAW 264.7 cells transfected with EGF-actin, has provided important information regarding the dynamics of the podosomes [20,21]. Firstly, a thin actin cloud surrounds the actin core of the podosome. Secondly, continuous actin turnover occurs in the actin core. Following this, the assembly pattern of podosomes self-organizes from the podosome cluster into the podosome ring and then to the podosome belt during differentiation. However, the origin of self-organization remains unknown. The detailed structure of the sealing zone was previously resolved by correlated fluorescence and high-resolution scanning electron microscopy [22]. The sealing zone consists of a dense array of actin cores connected via a network of F-actin, which corresponds to the thin actin cloud described above. The actin core contains F-actin and actin-associated proteins including Arp2/3, gelsolin, and cofilin. A ring-like adhesion domain surrounds each actin core of the podosome. The adhesion domain is also connected to the core via radial F-actin. The adhesion domain contains cell–matrix adhesion proteins, i.e., integrins, and their associated proteins, such as vinculin, paxillin, and zyxin. These observations establish the basis for understanding the behavior of the sealing zone during bone resorption.

### 3.2. The Sealing Zone on Bone Is Different from the Podosome Belt on Glass

The critical question is whether the sealing zone on bone is the same as the podosome belt, as seen on glass. A previously reported hypothesis denotes that the sealing zone of the bone comprises similar building units to the individual podosome that, however, differ in their density and degree of inter-connectivity compared to the podosome belt [22]. Yet, there are several differences between the two structures. Firstly, the characteristic actin core that is easily identifiable in the podosome belt is rarely seen in the sealing zone. Secondly, podosomes are a mechanosensitive structure. The formation of the podosome belt in osteoclasts depends on the physical and chemical properties of the substrate [23]. When osteoclasts are seeded on a hemislide with half the surface being coated with apatite and the other half being glass, the cells on apatite form the sealing zone, whereas those on glass develop the podosome belt [24]. Furthermore, the protrusion force generated by the actin core of the podosome changes depending on the substrate stiffness [25,26]. These results illustrate the extraordinary plasticity of podosomes in response to environmental cues. Accordingly, it is reasonable to deduce that the sealing zone and the podosome belt have differences. The conclusion also indicates the limit of in vitro observations. In vivo observations of the sealing zone would potentially reveal distinct organizations depending on the positional cues and the quality of bone. 

### 3.3. Size of the Sealing Zone

**Figure 2:**
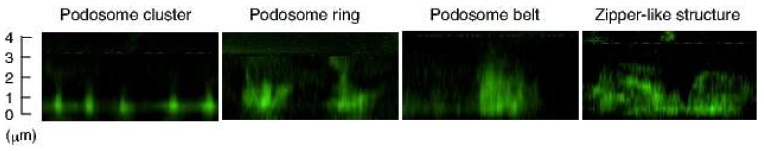
The height of F-actin-rich domains of the podosome-related structures of osteoclasts. Osteoclasts differentiated from RAW 264.7 cells on glass were stained with FITC (fluorescein isothiocyanate)-phalloidin for N-structured illumination microscopy (SIM) imaging. Z-stacks of the entire FITC-positive volume were acquired at 0.3 µm intervals. The images are representatives of cross sections of the podosome cluster, the podosome ring, the podosome belt, and the zipper-like structure.

At present, the standard spatial model of the podosomes has been constructed on the basis of observations of the podosome cluster formed in macrophages and dendritic cells on glass [3,27]. Does this model also apply for the sealing zone of osteoclasts? In this section of the study, we investigate the size of the sealing zone. In osteoclasts, the height of the phalloidin-positive F-actin-rich domain of the podosome cluster is 1 µm, whereas the height of the sealing zone is 4 µm [21]. Measurements using N-SIM (structured illumination microscopy) showed that the height of the F-actin-rich domain in osteoclasts derived from RAW264.7 cells on glass varies according to the following order: podosome cluster < the podosome ring < the podosome belt = the zipper-like structure (ZLS) (Figure 2) [6]. The gross morphology of the F-actin-rich domain in the first two structures appears as the actin core, whereas it is often difficult to recognize the actin core in the other two structures. In addition, there are variations in the area in which the phalloidin-positive F-actin-rich domain distributes. In the podosome cluster, the actin cores distribute in a specified area of the ventral membrane with a variable size. In contrast, the F-actin-rich domain is confined within a width of about 4 µm in the podosome ring, the podosome belt, the ZLS, and the sealing zone [6]. Furthermore, the spatial displacement of the actin core against the adhesion domain containing vinculin, paxillin, and zyxin is reasonably different from that in the podosome belt [28,29] or the ZLS [6]. So, the present spatial model for the podosome cluster does not match with that of the sealing zone. Future studies using super-resolution microscopy are needed to elucidate the organization of the sealing zone on bone.

## 4. Actin Flow

### 4.1. Discovery of Self-Organized Actin Flow in Osteoclasts

**Figure 3:**
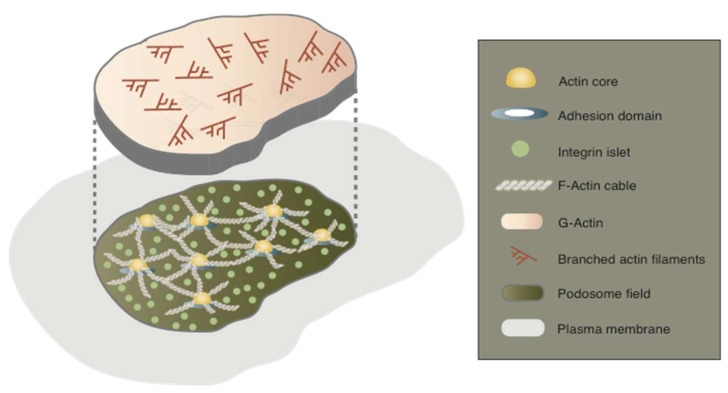
Model of the podosome field. The model illustrates a perspective of the basic structural elements of the podosome field in the pattern of the podosome cluster. The podosome field represents a distinct entity in the plasma membrane and comprises the actin cores, a network of F-actin cables, integrin islets, and the actin flow. The actin flow is generated by Arp2/3-dependent branched actin elongation. Pattern transitions of the podosome field may produce the podosome cluster, the podosome ring, the podosome belt, and the zipper-like structure in osteoclasts.

Plasticity and dynamics are vital for the function of the sealing zone, because osteoclasts attach on the rugged surface of the bone and segregate the area of bone resorption via the sealing zone. The dynamics of the podosome belt has repeatedly been investigated since its discovery [19,20,30]. However, it remains unknown how the podosome belt changes and maintains its gross shape. We have proposed that the actin flow drives the dynamics of the osteoclast podosomes [6,7]. Actin dynamics were examined by recording confocal images of the ZLS in RAW 264.7 cells transfected with EGFP (enhanced green fluorescent protein)–actin on glass. When two osteoclasts come into contact during cell fusion, the podosome belt transforms into the ZLS at the contact site [31,32]. The ZLS spans two cells and is probably involved in the juxtaposition of fusion partners. Movies reconstructed from images taken at 4 s intervals revealed the consistent retrograde flow of EGFP–actin in the ZLS. The mean flow rate was 3.32 ± 1.94 µm/min (mean ± S.D). Interestingly, the actin flow was observed in all the podosome-derived structures: the podosome cluster, the podosome ring, and the podosome belt. In these structures, the intensity of the signals from the actin flow was lower than that of the signals from the actin cores. The behavior of the actin flow was independent of the turnover of the actin cores. Actin flows in these structures were sporadic and multidirectional. Because the actin flow depends on Arp2/3 activity, it differs from the thin actin cloud that is composed of F-actin. Hence, actin flow appears to be a new element of the podosome-derived structures in osteoclasts. The most up-to-date model defines the podosme as a multifunctional zone comprising the actin cores, integrin islets, and a network of F-actin [27]. We added actin flow to this model and termed this multifunctional zone the “podosome field” (Figure 3) [6,7]. We assume that the podosome field is equal to an intracellular organelle, because it represents a distinct organization from the surroundings. The podosome field would, therefore, change its organization into the podosome ring, the podosome belt, or the sealing zone, as described below.

### 4.2. Actin Waves in Dictyostelium Cells and Actin Flow in Osteoclasts

**Figure 4:**
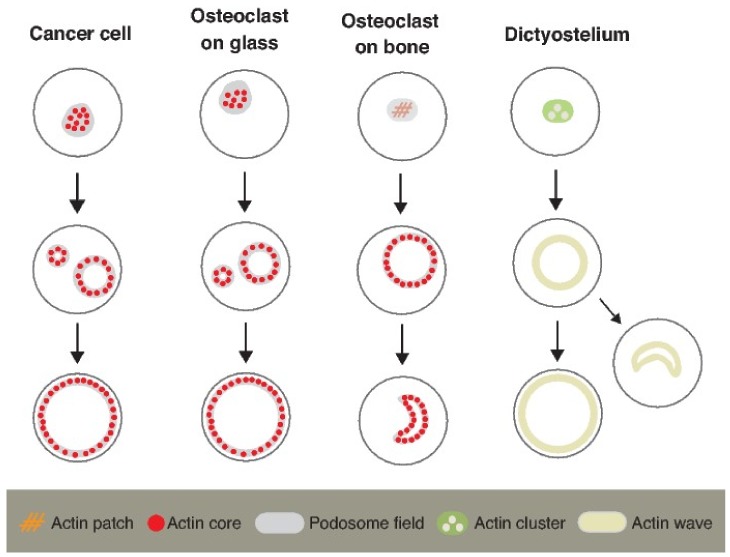
Pattern transition of actin assembly. A bird’s-eye view of the various actin assemblies. The assembly pattern of the actin core evolves from a cluster, to a ring, to a belt in invadopodia in RSV-BHK cells [28] and in osteoclasts on glass [21]. In osteoclasts on apatite–collagen-coated glass [24] and on dentin [38], a ring-like sealing zone evolves from the uncharacterized actin patch. Most crescent-like sealing zones (80%) on bone evolve from a ring-shaped sealing zone, whereas a small number of them (20%) directly develops from the actin patch [15]. In *Dictyostelium* cells, the actin wave takes various patterns including a circular ring, an arc shape, and a belt [39]. The actin wave originates from the actin clusters embedded in a PtdIns(3,4,5)P3 patch. The wave does not contain the actin core.

Before evaluating the role of the actin flow in the sealing zone, we will briefly describe the actin flow in other cells. The wave-like movement of actin and actin-associated proteins, often called the “ventral actin wave” or “traveling wave”, is observed in many cells [33,34]. Of these, we will introduce the actin wave of *Dictyostelium* cells. The actin wave is a self-organized structure driven by Arp2/3-dependent actin elongation [35,36,37]. The actin wave does not take a fixed shape. It can change the direction of propagation and can cause both fusion and fission. The actin wave develops from a local actin cluster and transforms it into a circular ring. The wave forms a lamellipodial structure at the cell periphery (Figure 4).

On the other hand, the formation of the sealing zone on apatite–collagen-coated glass [24] and on dentin [38] is preceded by the appearance of the actin patch, an uncharacterized actin cluster. In osteoclasts on glass, the podosome cluster develops into the podosome ring and the podosome belt [20]. A similar pattern transition is also observed in the invadopodia of Rous sarcoma virus (RSV)-transformed baby hamster kidney (BHK) cells [28]. The assembly of actin in osteoclasts and transformed cells is highly dynamic and often causes fusion and fission. Because these behaviors resemble those of the actin wave in *Dictyostelium*, we proposed that the transition of actin assembly in osteoclasts is caused by the actin flow in the podosome field [6,7].

### 4.3. The Behavior of the Actin Wavse in a Large Space

The fusion of mononuclear precursor cells produces a giant osteoclast in differentiation. The bigger the osteoclast, the more efficiently it resorbs bone [40]; however, it is unknown why bigger osteoclasts are more active. Is there a relationship between the formation of the sealing zone and the area of a multinucleated cell? Gerhard et al. fused mononuclear *Dictyostelium* cells together by electric pulses and compared the behavior of the actin waves in mononuclear cells to that in giant cells [39]. They concluded that a key principle of the produced wave patterns is an inherent length scale that does not vary with cell size. However, the circular actin wave in giant cells polarizes in two segments: a leading segment and a trailing segment. This polarization is often associated with a shape change from a circular ring to an arc-shaped wave. Therefore, the actin wave in a multinucleated cell exhibits more complex behavior that that in a mononuclear cell. This leads to a simple working model denoting that the formation of the sealing zone needs a large space in the ventral membrane, which is provided by the multinucleated cell. In giant *Dictyostelium* cells, the PtdIns(phosphatidylinositol) (3,4,5)P3-rich region between the leading and trailing waves is in an excited state [39]. We can speculate that the exocytic transport vesicles with H^+^-ATPase (V-ATPase) in this excited region lead to the establishment of the ruffled border membrane [41].

An early study recognized that, in vivo, rat osteoclasts formed ring- and arch-shaped sealing zones [42]. When osteoclasts resorb bone, they produce two types of resorption traces: a “pit”-type and “trail”- or “trench”-type (Figure 1). Human osteoclasts with a circular sealing zone form the resorption pit, whereas those with a crescent-shaped sealing zone produce trail- or trench-type resorption traces on dentin as well as on bone [14,15]. The pit-forming osteoclast attaches on a flat bone surface via a circular sealing zone and forms a pit by degrading bone under the ruffled border membrane. Osteoclasts that form trail- or trench-type resorption traces adhere to bone in a different manner. The leading segment of a crescent-shaped sealing zone attaches on the naive bone (outside of the digested cavity), whereas the trailing segment adheres to the “wall” of the existing digested cavity [14]. Such configuration of the sealing zone allows osteoclasts to vertically resorb bone and move horizontally at the same time. In a crescent-shaped sealing zone, the trailing segments are unstable compared to the leading segments [15]. The result is confirmed in the crescent-type podosome belt of RAW 264.7 cells on glass [43]. Interestingly, most trench-forming osteoclast (80%) are derived from pit-forming osteoclasts [15]. These results emphasize the plasticity and local dynamics of the sealing zone on bone. Does the sealing zone contribute to the movement of osteoclasts? We have previously reported that retrograde actin flow occurs in the ZLS, a local segment of the podosome belt in RAW 264.7 cells on glass [6]. It is well established that retrograde actin flow in the lamellipodia generates the driving force for fibroblast migration [44]. Accordingly, a possible scenario is that the movement of osteoclasts is driven by retrograde actin flow in the leading segment of a crescent-shaped sealing zone. Future studies are needed to ask whether the actin flow occurs in the sealing zone of osteoclasts on bone.

## 5. Organization of the Podosome Field 

Organelles result from the selective gathering and ordered disposition of intracellular materials in a cell. The sealing zone is often called a membrane-less organelle. To elucidate the organogenesis of the sealing zone, it would be useful to apply established knowledge from other fields. The principles that might be applicable here are protein condensation and self-organization.

### 5.1. Two Substructures of the Podosome Belt

There are many studies on the mechanism of podosomes/invadopodia formation. However, the main focus of these studies is the formation of the actin core with the adhesion ring. Here, we divide the podosome field into two actin-based substructures: the actin core with the adhesion ring, and the actin flow. The former is responsible for the generation of protrusions and mechanosensitivity, while the latter is responsible for pattern transition of the podosome field. The two structures can coexist in the podosome cluster, the podosome ring, and the podosome belt of osteoclasts [6]. Different regulatory systems organize the two actin substructures, as described below. Previous studies divided the podosome belt into the actin core and the actin cloud, a loose network of radial actin cables [20,45,46]. At present, there is no evidence that the actin cloud causes the actin flow, as described above. Two phenotypes of the podosome belt in osteoclasts are observed by suppressing the expression of a specific gene or functionally inhibiting the activity of a specific molecule: absence of an actin core with the morphology of the podosome belt and an actin core without the morphology of the podosome belt [46]. Osteoclasts that are deficient in gelsolin [47], Wiskott–Aldrich syndrome protein (WASP) [48], and WASP-interacting protein (WIP) [45] show the former phenotype. These molecules are distributed in the actin core and are involved in actin polymerization. The latter phenotype is observed in osteoclasts deficient in Src [46], Kindlin-3 [49], and those treated with inhibitors for dynamin and microtubules [43]. These proteins are localized both in and/or out of the actin core and not directly involved in actin polymerization. The results suggest that the formation of two substructures is separable, although they are both organized by Arp2/3-dependent actin elongation. Therefore, we will discuss the formation of the actin ring and the actin flow separately in the following sections. 

### 5.2. Formation of Actin Core with the Adhesion Domain

The mechanism of actin core formation in the podosome cluster has been well studied [50]. Normal fibroblasts form focal adhesions on glass coated with Arg–Gly–Asp (RGD) peptide, but form podosome-like structures when plated on fluid RGD-lipid surfaces, a condition where there is no traction force between the plasma membrane and the substrate. On freely diffusible RGD lipids, fibroblasts first form integrin clusters. The clusters become enriched with PtdIns(3,4,5)P3, which is followed by the recruitment of adaptor proteins, such as talin and paxillin, and regulatory proteins for actin polymerization. The activation of N-WASP drives the Arp2/3-dependent actin polymerization. The integrin clusters finally transform into the actin core with the adhesion ring by the depletion of the RGD core at the center of the cluster. Key to this organogenesis is the ligand-dependent integrin clustering in the absence of traction force. At least two factors are needed for the clustering of integrins in this case. In osteoclasts, the podosome cluster appears on glass without the ligand. Furthermore, osteoclasts can form the podosome belt on dentin, but not on demineralized dentin [23,24]. These results suggest that multiple factors, such as the rigidity of the matrix, soluble factors, and integrin signaling, are probably involved in the initiation of integrin clustering in osteoclasts.

The emerging paradigm of cellular organogenesis is protein condensation [51,52]. When proteins in solution are condensed, they enter into different physical states, such as dense liquid, filament, gel, and glass. These condensates establish the membrane-less compartments that allow for the specific enrichment of certain molecules. There are several requirements for the appearance of such compartments. They include a nucleator that initiates the condensation, a multivalent protein that drives the assembly of interacting macromolecules, and a polymer-like behavior that generates the different dynamics and morphologies. If the actin core with the adhesion domain is organized on the basis of this paradigm, the initiator of the organization might be the condensation of integrins, a phenomenon in the equilibrium state.

### 5.3. Formation of the Actin Flow

Computational modeling suggests that the Arp2/3-dependent actin treadmilling produces the actin patches and the actin waves in a stochastic manner [53]. In this model, existing actin filaments are required to attach to the membrane. The branched elongation of actin filaments from the existing filaments is modulated by positive feedback, spreading, and delayed negative feedback mechanisms. In the self-organized system, a small change in the boundary conditions, such as a change in the relative level of actin, Arp2/3, or capping protein, produces a drastic pattern change of assembly of actin filaments in both computational [53] and in vitro systems [54,55]. Even surface geometry changes the rate of Arp2/3-dependent actin polymerization and the motility of reconstituted Listeria [56]. Traditional studies have focused on finding the signature molecules for each assembly pattern of podosomes. However, such efforts might not produce fruitful results in the self-organized system.

Self-organization is a characteristic of a dissipative structure in the non-equilibrium state [57]. Actin polymerization in the actin flow of the podosome field must be maintained by the energy released from hydrolysis of ATP supplied from the cytoplasm. The wave behavior of the actin flow ensures the flexibility and dynamics of the podosome field. Because polymerization of G-actin increases the viscosity, the diffusion of molecules becomes restricted in the podosome field. There is evidence that the actin wave is involved in organogenesis. The actin wave in *Dictyostelium* cells transforms into the 2D-phagocytic cup, mimicking the phagocytic cup during phagocytosis of macrophages [58]. The 2D-phagocytic cup is functional: it can take up a particle of heat-killed Saccaromyces cerevisiae.

In summary, the formation of the podosome field results from the coupling of the actin core with the adhesion domain and the self-organized actin wave. Each substructure appears to originate from the organizational principle at equilibrium or non-equilibrium state. In future studies, hopefully, these features would help to elucidate the formation and behavior of the sealing zone on bone.

## 6. Functions of the Sealing Zone

### 6.1. Diffusion Barrier

During bone resorption, osteoclasts prevent the free diffusion of proteins, proteases, and acid from the resorption lacune. Accordingly, osteoclasts firmly attached on plastic can create a microenvironment underneath the cell at a pH value of less than 3 [59]. Lucifer yellow (MW = 480 Da) cannot penetrate underneath the osteoclasts on glass [60]. Amazingly, the general cell surface biotinylation reagent sulfo-*N*-hydroxysuccinimide (MW = 115 Da) does not label membrane proteins in podosome-derived structures of osteoclasts on glass [31]. Many researchers believe that the sealing zone or the podosome belt functions as a diffusion barrier for ions, lipids, and proteins, similar to the tight junctions of epithelia [60]. Another proposed mechanism suggests that the concentration of a given molecule in the resorption lacune derives from a balance between the generation rate and its limited diffusion, rather than from the presence of the diffusion barrier [61]. We will discuss this issue in terms of membrane compartmentalization below.

The polarization of osteoclasts divides osteoclasts’ ventral membrane into three domains; the sealing zone, the ruffled border membrane, and the remaining ventral plasma membrane (Figure 1). Peanut agglutinin (PNA) specifically binds to the ruffled border membrane [62], whereas VSV-G-proteins are distributed in the ventral plasma membrane [63]. The sealing zone is negative for both PNA and VSV-G-protein. These results clearly indicate that the sealing zone blocks the lateral diffusion of membrane proteins.

In macrophages, diffusion of PtdIns(4,5)P2 and plasma membrane-anchored GFP from the phagocytic cup is impeded during phagocytosis [64]. Similarly, a membrane-type phosphatase, CD45, is excluded from the phagocytic cup [65]. This exclusion is due to the generation of an expanding diffusion barrier triggered by integrin activation. Given that the phagocytic cup of macrophages is a type of pododosme field, as discussed above, integrins in the sealing zone might also play a key role in forming the diffusion barrier in osteoclasts. In this regard, it is interesting that a low dose of echistatin, an RGD-containing disintegrin, causes the dispersion of the polarized distribution of α_v_β3 integrin in the sealing zone and inhibits bone resorption of osteoclasts on dentin [66]. As discussed above, integrin islets are distinct components of the podosome field (Figure 3). The clustering of integrin plays a pivotal role in the formation of the actin core with an adhesion domain (Section 5.2). Curiously, the actin core spontaneously appears and disappears in all areas of the podosome field. Because integrin should link the plasma membrane to the extracellular matrix, an uncharacterized assembly pattern of integrin molecules may be involved in the barrier function of the sealing zone. Future studies are needed to elucidate the behavior of integrins, especially at the level of a single molecule, to substantiate the molecular mechanisms of diffusion barrier function in osteoclasts.

### 6.2. Matrix Degradation

Bone resorption is associated with the compartmentalization of the ruffled border membrane [67]. In the stationary osteoclasts with a ring-shaped sealing zone on bone, the transport of exocytic vesicles delivers V-ATPase and cathepsin K to the peripheral “fusion zone” of the ruffled border membrane. The fusion zone is enriched with lipid rafts, which are a type of cholesterol/ganglioside-rich membrane domains. In contrast, degraded bone materials are endocytosed from the center of the ruffled border membrane, called the “uptake zone”. Hence, the ruffled border membrane is the active site of bone resorption. It is noteworthy that the ruffled border membrane has a protrusive morphology, i.e., it protrudes into the bone. On the other hand, the sealing zone contains another pH regulator, namely, Na^+^-H^+^ exchanger 1 (NHE-1) [68]. However, this exchanger is not involved in the acidification of the resorption lacune [69].

In osteoclasts with a crescent-shaped sealing zone on bone, the “fusion zone” and “uptake zone” are arranged along the axis of cell movement. The “fusion zone” is located at the leading edge of the ruffled border membrane that faces the leading segment of a crescent-shaped sealing zone [67,70]. The trailing segment of the sealing zone runs across the “uptake zone”. Thus, endocytosis occurs both inside and outside the trailing segment of the sealing zone. Of note, it is often difficult to discern the trailing segment of a crescent-shaped sealing zone. This probably suggests the degradation of the normal organization of actin filaments in the trailing segment. Such local dynamics of the sealing zone may produce osteoclasts characterized by a “line-shaped” sealing zone in rat bone [42]. Furthermore, osteoclasts with a crescent-shaped sealing zone show higher collagenolytic activity and form deeper resorption traces than those with a ring-shaped sealing zone [70]. These results suggest that both bone-resorbing osteoclasts in the stationary and migratory modes form the specialized domains in the ventral membrane, but the arrangements of these organelles are quite different. In particular, because the endocytosis domain spreads out of the sealing zone in osteoclasts in the migratory mode, we may have to revise the definition of the ruffled border membrane in the future.

Osteoclasts plated on a plastic dish form a podosome belt but not a ruffled border membrane [23]. However, osteoclasts on a plastic dish establish an acidic environment between the ventral membrane and the culture dish [59]. The podosome belt of osteoclasts can digest gelatin coated with vitronectin on glass [28,46]. Thus, osteoclasts on plastic or on glass secrete protons and proteases in the absence of the ruffled border membrane. The mechanosensitivity of the podosome field probably results in the apparent contradictory observations. In response to cues presented by the experimental conditions, osteoclasts might exhibit a different degree of polarization, that is, a difference in membrane compartmentalization and intracellular traffic pathways. It would be interesting to examine whether the sealing zone has the ability to digest some components of bone in future investigations.

It is widely accepted that invadopodia are a site of matrix degradation [1,71]. In MDA-MB-231 cells, NHE-1 in the invadopodia acidifies the peri-invadopodial space, resulting in the activation of proteases [72,73,74]. Do invadopodia have specialized subdomains, similar to the ruffled border membrane of osteoclasts? NHE-1 with its regulatory proteins, CD44 and hyaluronidase-2, is enriched in lipid rafts in MDA-MB-231 cells [72]. In Src-transformed NIH cells, scaffolding proteins, including liprin-α1, ERC1, and LLT, are localized in the plasma membrane-associated platform (PMAP) next to the core of invadopodia and are involved in migration and matrix degradation [75,76]. These results suggest that the mechanism of matrix degradation by invasive cells differs from that of bone degradation by osteoclasts. However, both cell types adopt a similar strategy for matrix digestion by generating specialized subdomains in their plasma membranes.

## 7. Perspectives

Recent advances in cell biology with high-resolution imaging and computational modeling have provided us with novel knowledge concerning the behavior of the cortical actin cytoskeleton. Especially, the knowledge of podosome dynamics helps us to understand the formation, dynamics, and structure of the sealing zone of osteoclasts. Here, we introduced the significance of the actin flow in the podosome field. The actin flow may resolve the unsettled issue of the pattern transition of actin assembly in podosomes/invadopodia and in the sealing zone in osteoclasts. Bone-resorbing osteoclasts can be divided at least in two types: osteoclasts in a stationary mode with a ring-shaped sealing zone and osteoclasts in a migratory mode with a crescent-shaped sealing zone. In the migratory osteoclasts, the leading segment of a crescent-shaped sealing zone behaves differently from the trailing segment of the sealing zone. Retrograde actin flow in the leading segment of a crescent-shaped sealing zone may explain the mechanism of movement during bone resorption. The formation of the actin core begins with integrin condensation and the successive recruitment of heterogeneous proteins, resulting in the organization of actin polymerization. The podosome field has two self-organized systems that depend on actin polymerization: the actin core and the actin flow. It would be instrumental to temporarily assign a distinct function to each substructure of the podosome field. The actin flow is involved in the formation and dynamics of the sealing zone. The actin core is responsible for the protrusive behavior of the sealing zone. The adhesion domain contains sensor proteins that sense the stiff geographical landscape of the bone. Integrins form a diffusion barrier for the sealing of the resorption lacune. Interconnected F-actin cables contribute to the collective behavior of the sealing zone. These functional classifications could be tested at least in in vitro experiments. The establishment of the sealing zone appears to cause the formation of the ruffled border membrane. Cues presented by the bone may stimulate the subdivision of the ruffled border membrane. How the sealing zone controls the formation of the ruffled border membrane is an issue for future research. Such challenges would be useful to deepen our understanding of the mechanisms of bone resorption by osteoclasts and may lead to the development of novel therapeutics to treat bone-related diseases.

## References

[1] Linder S., Wiesner C., Himmel M. (2011). Degrading devices: Invadosomes in proteolytic cell invasion. Annu. Rev. Cell Dev. Biol..

[2] Murphy D.A., Courtneidge S.A. (2011). The “ins” and “outs” of podosomes and invadopodia: Characteristics, formation and function. Nat. Rev. Mol. Cell Biol..

[3] Albiges-Rizo C., Destaing O., Fourcade B., Planus E., Block M.R. (2009). Actin machinery and mechanosensitivity in invadopodia, podosomes and focal adhesions. J. Cell Sci..

[4] Linder S., Wiesner C. (2016). Feel the force: Podosomes in mechanosensing. Exp. Cell Res..

[5] Di Martino J., Henriet E., Ezzoukhry Z., Goetz J.G., Moreau V., Saltel F. (2016). The microenvironment controls invadosome plasticity. J. Cell Sci..

[6] Takito J., Otsuka H., Inoue S., Kawashima T., Nakamura M. (2017). Symmetrical retrograde actin flow in the actin fusion structure is involved in osteoclast fusion. Biol. Open.

[7] Takito J., Inoue S., Nakamura M. (2017). Emerging role of actin flow in the organization of podosomes in osteoclasts. Macrophage.

[8] Teti A., Marchisio P.C., Zallone A.Z. (1991). Clear zone in osteoclast function: Role of podosomes in regulation of bone-resorbing activity. Am. J. Physiol..

[9] Väänänen H.K., Zhao H., Mulari M., Halleen J.M. (2000). The cell biology of osteoclast function. J. Cell Sci..

[10] Baron R., Neff L., Louvard D., Courtoy P.J. (1985). Cell-mediated extracellular acidification and bone resorption: Evidence for a low pH in resorbing lacunae and localization of a 100-kD lysosomal membrane protein at the osteoclast ruffled border. J. Cell Biol..

[11] Blair H.C., Teitelbaum S.L., Ghiselli R., Gluck S. (1989). Osteoclastic bone resorption by a polarized vacuolar proton pump. Science.

[12] Nesbitt S.A., Horton M.A. (1997). Trafficking of matrix collagens through bone-resorbing osteoclasts. Science.

[13] Salo J., Lehenkari P., Mulari M., Metsikkö K., Väänänen H.K. (1997). Removal of osteoclast bone resorption products by transcytosis. Science.

[14] Rumpler M., Wurger T., Roschger P., Zwettler E., Sturmlechner I., Altmann P., Fratzl P., Rogers M.J., Klaushofer K. (2013). Osteoclasts on bone and dentin in vitro: Mechanism of trail formation and comparison of resorption behavior. Calcif. Tissue Int..

[15] Søe K., Delaissé J.M. (2017). Time-lapse reveals that osteoclasts can move across the bone surface while resorbing. J. Cell Sci..

[16] Soriano P., Montgomery C., Geske R., Bradley A. (1991). Targeted disruption of the c-src proto-oncogene leads to osteopetrosis in mice. Cell.

[17] Boyce B.F., Yoneda T., Lowe C., Soriano P., Mundy G.R. (1992). Requirement of pp60c-src expression for osteoclasts to form ruffled borders and resorb bone in mice. J. Clin. Investig..

[18] Schenk R.K., Spiro D., Wiener J. (1967). Cartilage resorption in the tibial epiphyseal plate of growing rats. J. Cell Biol..

[19] Marchisio P.C., Cirillo D., Naldini L., Primavera M.V., Teti A., Zambonin-Zallone A. (1984). Cell-substratum interaction of cultured avian osteoclasts is mediated by specific adhesion structures. J. Cell Biol..

[20] Destaing O., Saltel F., Geminard J.C., Jurdic P., Bard F. (2003). Podosomes display actin turnover and dynamic self-organization in osteoclasts expressing actin-green fluorescent protein. Mol. Biol. Cell.

[21] Jurdic P., Saltel F., Chabadel A., Destaing O. (2006). Podosome and sealing zone: Specificity of the osteoclast model. Eur. J. Cell Biol..

[22] Luxenburg C., Geblinger D., Klein E., Anderson K., Hanein D., Geiger B., Addadi L. (2007). The architecture of the adhesive apparatus of cultured osteoclasts: From podosome formation to sealing zone assembly. PLoS ONE.

[23] Nakamura I., Takahashi N., Sasaki T., Jimi E., Kurokawa T., Suda T. (1996). Chemical and physical properties of the extracellular matrix are required for the actin ring formation in osteoclasts. J. Bone Miner. Res..

[24] Saltel F., Destaing O., Bard F., Eichert D., Jurdic P. (2004). Apatite-mediated actin dynamics in resorbing osteoclasts. Mol. Biol. Cell.

[25] Collin O., Na S., Chowdhury F., Hong M., Shin M.E., Wang F., Wang N. (2008). Self-organized podosomes are dynamic mechanosensors. Curr. Biol..

[26] Labernadie A., Bouissou A., Delobelle P., Balor S., Voituriez R., Proag A., Fourquaux I., Thibault C., Vieu C., Poincloux R. (2014). Protrusion force microscopy reveals oscillatory force generation and mechanosensing activity of human macrophage podosomes. Nat. Commun..

[27] Van den Dries K., Schwartz S.L., Byars J., Meddens M.B., Bolomini-Vittori M., Lidke D.S., Figdor C.G., Lidke K.A., Cambi A. (2013). Dual-color superresolution microscopy reveals nanoscale organization of mechanosensory podosomes. Mol. Biol. Cell.

[28] Badowski C., Pawlak G., Grichine A., Chabadel A., Oddou C., Jurdic P., Pfaff M., Albiges-Rizo C., Block M.R. (2008). Paxillin phosphorylation controls invadopodia/podosomes spatiotemporal organization. Mol. Biol. Cell.

[29] Ory S., Brazier H., Pawlak G., Blangy A. (2008). Rho GTPases in osteoclasts: Orchestrators of podosome arrangement. Eur. J. Cell Biol..

[30] Kanehisa J., Yamanaka T., Doi S., Turksen K., Heersche J.N., Aubin J.E., Takeuchi H. (1990). A band of F-actin containing podosomes is involved in bone resorption by osteoclasts. Bone.

[31] Takito J., Nakamura M., Yoda M., Tohmonda T., Uchikawa S., Horiuchi K., Toyama Y., Chiba K. (2012). The transient appearance of zipper-like actin superstructures during the fusion of osteoclasts. J. Cell Sci..

[32] Takito J., Nakamura M. (2012). Precursors linked via the zipper-like structure or the filopodium during the secondary fusion of osteoclasts. Commun. Integr. Biol..

[33] Allard J., Mogilner A. (2013). Traveling waves in actin dynamics and cell motility. Curr. Opin. Cell Biol..

[34] Inagaki N., Katsuno H. (2017). Actin Waves: Origin of Cell Polarization and Migration?. Trends Cell Biol..

[35] Bretschneider T., Anderson K., Ecke M., Muller-Taubenberger A., Schroth-Diez B., Ishikawa-Ankerhold H.C., Gerisch G. (2009). The three-dimensional dynamics of actin waves, a model of cytoskeletal self-organization. Biophys. J..

[36] Schroth-Diez B., Gerwig S., Ecke M., Hegerl R., Diez S., Gerisch G. (2009). Propagating waves separate two states of actin organization in living cells. HFSP J..

[37] Gerisch G., Ecke M., Wischnewski D., Schroth-Diez B. (2011). Different modes of state transitions determine pattern in the Phosphatidylinositide-Actin system. BMC Cell Biol..

[38] McMichael B.K., Cheney R.E., Lee B.S. (2010). Myosin X regulates sealing zone patterning in osteoclasts through linkage of podosomes and microtubules. J. Biol. Chem..

[39] Gerhardt M., Ecke M., Walz M., Stengl A., Beta C., Gerisch G. (2014). Actin and PIP3 waves in giant cells reveal the inherent length scale of an excited state. J. Cell Sci..

[40] Yagi M., Miyamoto T., Sawatani Y., Iwamoto K., Hosogane N., Fujita N., Morita K., Ninomiya K., Suzuki T., Miyamoto K. (2005). DC-STAMP is essential for cell–cell fusion in osteoclasts and foreign body giant cells. J. Exp. Med..

[41] Palokangas H., Mulari M., Väänänen H.K. (1997). Endocytic pathway from the basal plasma membrane to the ruffled border membrane in bone-resorbing osteoclasts. J. Cell Sci..

[42] Kuroda H., Nakamura M., Kamiyama K. (1996). Effects of calcitonin and parathyroid hormone on the distribution of F-actin in the clear zone of osteoclasts in vivo. Bone.

[43] Batsir S., Geiger B., Kam Z. (2017). Dynamics of the sealing zone in cultured osteoclasts. Cytoskeleton.

[44] Pollard T.D., Borisy G.G. (2003). Cellular motility driven by assembly and disassembly of actin filaments. Cell.

[45] Chabadel A., Banon-Rodriguez I., Cluet D., Rudkin B.B., Wehrle-Haller B., Genot E., Jurdic P., Anton I.M., Saltel F. (2007). CD44 and β3 integrin organize two functionally distinct actin-based domains in osteoclasts. Mol. Biol. Cell.

[46] Saltel F., Chabadel A., Bonnelye E., Jurdic P. (2008). Actin cytoskeletal organisation in osteoclasts: A model to decipher transmigration and matrix degradation. Eur. J. Cell Biol..

[47] Chellaiah M., Kizer N., Silva M., Alvarez U., Kwiatkowski D., Hruska K.A. (2000). Gelsolin deficiency blocks podosome assembly and produces increased bone mass and strength. J. Cell Biol..

[48] Calle Y., Jones G.E., Jagger C., Fuller K., Blundell M.P., Chow J., Chambers T., Thrasher A.J. (2004). WASp deficiency in mice results in failure to form osteoclast sealing zones and defects in bone resorption. Blood.

[49] Schmidt S., Nakchbandi I., Ruppert R., Kawelke N., Hess M.W., Pfaller K., Jurdic P., Fässler R., Moser M. (2011). Kindlin-3-mediated signaling from multiple integrin classes is required for osteoclast-mediated bone resorption. J. Cell Biol..

[50] Yu C.H., Rafiq N.B., Krishnasamy A., Hartman K.L., Jones G.E., Bershadsky A.D., Sheetz M.P. (2013). Integrin-matrix clusters form podosome-like adhesions in the absence of traction forces. Cell Rep..

[51] Alberti S. (2017). The wisdom of crowds: Regulating cell function through condensed states of living matter. J. Cell Sci..

[52] Shin Y., Brangwynne C.P. (2017). Liquid phase condensation in cell physiology and disease. Science.

[53] Carlsson A.E. (2010). Dendritic actin filament nucleation causes traveling waves and patches. Phys. Rev. Lett..

[54] Kawska A., Carvalho K., Manzi J., Boujemaa-Paterski R., Blanchoin L., Martiel J.L., Sykes C. (2012). How actin network dynamics control the onset of actin-based motility. Proc. Natl. Acad. Sci. USA.

[55] Loisel T.P., Boujemaa R., Pantaloni D., Carlier M.F. (1999). Reconstitution of actin-based motility of Listeria and Shigella using pure proteins. Nature.

[56] Bernheim-Groswasser A., Wiesner S., Golsteyn R.M., Carlier M.F., Sykes C. (2002). The dynamics of actin-based motility depend on surface parameters. Nature.

[57] Glansdorff P., Prigogine I. (1978). Thermodynamic Theory of Structure, Stability and Fluctuations.

[58] Gerisch G., Ecke M., Schroth-Diez B., Gerwig S., Engel U., Maddera L., Clarke M. (2009). Self-organizing actin waves as planar phagocytic cup structures. Cell Adhes. Migr..

[59] Silver I.A., Murrills R.J., Etherington D.J. (1988). Microelectrode studies on the acid microenvironment beneath adherent macrophages and osteoclasts. Exp. Cell Res..

[60] Väänänen H.K., Horton M. (1995). The osteoclast clear zone is a specialized cell-extracellular matrix adhesion structure. J. Cell Sci..

[61] Stenbeck G., Horton M.A. (2000). A new specialized cell-matrix interaction in actively resorbing osteoclasts. J. Cell Sci..

[62] Takagi M., Yagasaki H., Baba T., Baba H. (1988). Ultrastructural visualization of selective peanut agglutinin binding sites in rat osteoclasts. J. Histochem. Cytochem..

[63] Salo J., Metsikkö K., Palokangas H., Lehenkari P., Väänänen H.K. (1996). Bone-resorbing osteoclasts reveal a dynamic division of basal plasma membrane into two different domains. J. Cell Sci..

[64] Golebiewska U., Kay J.G., Masters T., Grinstein S., Im W., Pastor R.W., Scarlata S., McLaughlin S. (2011). Evidence for a fence that impedes the diffusion of phosphatidylinositol 4,5-bisphosphate out of the forming phagosomes of macrophages. Mol. Biol. Cell.

[65] Freeman S.A., Goyette J., Furuya W., Woods E.C., Bertozzi C.R., Bergmeier W., Hinz B., van der Merwe P.A., Das R., Grinstein S. (2016). Integrins Form an Expanding Diffusional Barrier that Coordinates Phagocytosis. Cell.

[66] Nakamura I., Pilkington M.F., Lakkakorpi P.T., Lipfert L., Sims S.M., Dixon S.J., Rodan G.A., Duong L.T. (1999). Role of α(v)β(3) integrin in osteoclast migration and formation of the sealing zone. J. Cell Sci..

[67] Mulari M.T., Zhao H., Lakkakorpi P.T., Väänänen H.K. (2003). Osteoclast ruffled border has distinct subdomains for secretion and degraded matrix uptake. Traffic.

[68] Gupta A., Edwards J.C., Hruska K.A. (1996). Cellular distribution and regulation of NHE-1 isoform of the NA-H exchanger in the avian osteoclast. Bone.

[69] Henriksen K., Sørensen M.G., Jensen V.K., Dziegiel M.H., Nosjean O., Karsdal M.A. (2008). Ion transporters involved in acidification of the resorption lacuna in osteoclasts. Calcif. Tissue Int..

[70] Merrild D.M., Pirapaharan D.C., Andreasen C.M., Kjærsgaard-Andersen P., Møller A.M., Ding M., Delaissé J.M., Søe K. (2015). Pit- and trench-forming osteoclasts: A distinction that matters. Bone Rep..

[71] Chen W.T. (1989). Proteolytic activity of specialized surface protrusions formed at rosette contact sites of transformed cells. J. Exp. Zool.

[72] Bourguignon L.Y., Singleton P.A., Diedrich F., Stern R., Gilad E. (2004). CD44 interaction with Na^+^–H^+^ exchanger (NHE1) creates acidic microenvironments leading to hyaluronidase-2 and cathepsin B activation and breast tumor cell invasion. J. Biol. Chem..

[73] Busco G., Cardone R.A., Greco M.R., Bellizzi A., Colella M., Antelmi E., Mancini M.T., Dell’Aquila M.E., Casavola V., Paradiso A. (2010). NHE1 promotes invadopodial ECM proteolysis through acidification of the peri-invadopodial space. FASEB J..

[74] Greco M.R., Antelmi E., Busco G., Guerra L., Rubino R., Casavola V., Reshkin S.J., Cardone R.A. (2014). Protease activity at invadopodial focal digestive areas is dependent on NHE1-driven acidic pHe. Oncol. Rep..

[75] Astro V., de Curtis I. (2015). Plasma membrane-associated platforms: Dynamic scaffolds that organize membrane-associated events. Sci. Signal..

[76] Sala K., Raimondi A., Tonoli D., Tacchetti C., de Curtis I. (2018). Identification of a membrane-less compartment regulating invadosome function and motility. Sci. Rep..

